# Supplementary material for: Contribution of self- and other-regarding motives to (dis)honesty
Source: Sci Rep. 2020 Sep 28;10:15844. doi: 10.1038/s41598-020-72255-5 (PMC7522268; doi:10.1038/s41598-020-72255-5)

Contribution of self- and other-regarding motives to (dis)honesty

(Supplementary materials)

*Anastasia Shuster[1,2] and Dino J Levy[1,2]

[1]*Sagol School of Neuroscience, Tel Aviv University*

[2]*Coller School of Management, Tel Aviv University*

(*) Corresponding author

Corresponding Author contact information:

Phone: +1 212-585-54664

Email: Anastasia.Shuster@mssm.edu

Supplementary materials: Analyses

Interaction between value to self and value to other

To test if there is an interaction effect between ΔV_{self} and $\Delta V_{other,}$ we ran an additional regression model, that included the interaction term $\Delta V_{self} \times \Delta V_{other}$. The addition of the interaction term did not have a substantial influence on the self-interest and regard-for-others coefficients. Both coefficients remained significant in most participants (see Table S2) and uncorrelated across subjects (r(26)=0.16, p=0.41). More importantly, the coefficients are highly correlated between before and after the addition of the interaction term (self-interest: r(26)=0.96, p<0.001; regard-for-others: r(26)=0.95, p<0.001). the interaction term itself was non-significant (p>0.05) in 22 participants (79%), and was only slightly significant (0.05>p>0.01) in an additional 5 participants (18%; see Table S2).

Examining the role of efficiency and inequality

In the payoff structure used in this study, inequality and efficiency cannot be adequately disentangled from value to self (ΔV_{self}) and value to other (ΔV_{other}), as they are highly correlated with them. Specifically, both efficiency (i.e., total payoffs compared across options, ($\$self_{lie}$ + $\$other_{lie}$) / ($\$self_{truth}$ + $\$other_{truth}$)) and inequality (i.e., the difference in payoffs compared across options; ($\$self_{lie}$ - $\$other_{lie}$) / ($\$self_{truth}$- $\$other_{truth}$)) are correlated with value to other (r(38)=0.48, p=0.001 and r(38)=-0.76, p<0.001, respectively). Efficiency is also correlated with value to self (r(38)=0.71, p<0.001).

Thus, adding these parameters as predictors to the regression resulted in all four coefficients being insignificant for most participants (see Table S3). However, a model containing efficiency, inequality, value to self, and value to other, performed significantly better than a model containing only efficiency and inequality (t(27)=-3.32, p=0.002, two-tailed paired t-test between the models' r-squared scores). This suggests that there is a role for value to self and value to other over and above efficiency and inequality.

An additional step we took to explore the effects of efficiency and inequality on choice, was to construct less traditional measures for efficiency and inequality, such that would reduce the collinearity in the regression. For efficiency, we were able to eliminate the correlation with value to self, and reduce the correlation with value to other to r=0.46, p=0.002 by looking at the total payoffs in the Lie option only (efficiency' = $\$self_{lie}$ + $\$other_{lie}$). For inequality, we reduced the correlation with value to other to r=0.38, p=0.04 by computing it as a ratio between self and other, compared across options (inequality' = ($\$self_{lie}$/$\$other_{lie}$) / ($\$self_{truth}$/$\$other_{truth}$)). We then conducted another regression analysis using these revised parameters (efficiency' and inequality'), with and without ΔV_{self} and ΔV_{other}. The results of these regressions show two things. One, the full model again outperformed a model containing only efficiency' and inequality' (t(27)=-14.5, p<0.001). Two, self-interest and regard-for-others in the full model are: (1) significant for most participants (see Table S3), (2) uncorrelated (r(26)=-0.05, p=0.77), (3) highly correlated with the coefficients of the original regression (self-interest: r(26)=0.94, p<0.001, regard-for-others: r(26)=0.91, p<0.001).

Logistic regression of choice

To test whether our main finding holds when using a logistic regression approach, we modelled each choice separately as a binary regressor (0: choosing Truth, 1: choosing Lie), without averaging across repetitions. This approach yielded comparable results to the linear regression. That is, both coefficients remained significant in most participants and uncorrelated across subjects ($r(26)=-0.008$, $p=0.96$). Moreover, the coefficients from the linear regression and those from the logistic regression are highly correlated (self: $r=0.67$, $p<0.001$ and other: $r=0.59$, $p<0.005$). Therefore, we can conclude that for our data, running either a logistic regression on the actual choice data or a linear regression on the averaged data across repetitions yields similar results. As explained the main text, the reason we chose to use the linear regression in our main fMRI analyses is that it allows for a better and a clearer interpretation of participants' behavior. The linear coefficient represents the slope in the relationship between payoffs and choice. In other words, it is the *sensitivity to change in payoffs*, which is how we conceptualize self-interest and regard-for-others in our paper.

Controlling for door position and time in experiment

It is important to ensure that other than the payoffs, there are no alternative explanations for the observed variance in (dis)honest choices. For example, if participants have a bias toward a certain door location, or if they tend to become more dishonest as time goes by.

We minimized this concern in two ways, one rooted in the task's design and the other in the analysis procedure. First, the payoffs on each trial were assigned doors at random, to avoid introducing a potential bias. Second, because the dependent variable in the regression analysis is *average choice* across repetitions, and the doors' locations were random, there could not be a systematic link between choice, payoffs, and door positions. This is also true for the ongoing duration of the experiment, because the same payoffs were encountered at four different time points during the experiment.

Notwithstanding, to rigorously examine if door position and ongoing duration of the experiment might systematically affect choice, we added them as predictors to a logistic regression on choice, where each repetition of the same payoff is modelled separately (see Logistic Regression of Choice section for details). Door position was entered as a dummy variable, and time in the experiment as the trial number (1-160). These predictors had little effect on participants' behavior. The *door* dummy predictors were non-significant ($p>0.05$) for most participants (>82%), and slightly significant ($0.05>p>0.01$) for a small subset of participants (<14%). Similarly, the time predictor was not significant for 24 participants (86%), and slightly significant for only 3 participants (11%). Finally, even after introducing these control predictors, the self-interest and regard-for-others coefficients remained significant in most participants (96% and 89%, respectively). Moreover, they were still uncorrelated ($r(26)=-0.06$, $p=0.73$) across subjects, and these updated coefficients are highly correlated with the original coefficients that we estimated without entering the door position and time during the experiment into the model ($r=0.64$ and $r=0.54$, respectively). Therefore, we conclude that door position and time in the experiment did not play a significant role in participants' choices.

Gender differences

We tested if there are gender differences in several measures using two-sample two-tailed t-tests, and found none.

First, there were no differences in any of the coefficients – neither self-interest (M_{male}=0.17±0.08, M_{female}=0.17±0.05; t(26)=-0.5,p=0.61) nor regard-for-others (M_{male}=-0.16±0.07, M_{female}=-0.17±0.05; t(26)=-0.91,p=0.36). Second, there were no differences in reaction times – neither in the average time it took to complete a trial (M_{male}=2.8±0.5, M_{female}=2.8±0.5; t(26)=0.24, p=0.8), nor in the difference between average lie trials' RT and truth trials' RT (M_{male}=0.1±0.42, M_{female}=0.1±0.36; t(26)=-0.001, p=0.99).

Role of cognitive control in LPFC activity

A potential explanation to negative relationship between value to self and the LPFC comes from the role of the LPFC in cognitive control (Badre and Nee 2018) – participants might experience more conflict and need for control when the potential reward from lying ($\$Self_{Lie}$-$\$Self_{Truth}$) is small. If this was the case, we would expect to find a negative relationship between the potential reward from lying and reaction times, such that smaller differences would yield longer reaction times. Because our neural model controls for the effects of reaction times on value representation, it is a less plausible explanation for the observed result. Nonetheless, we directly tested this hypothesis.

To examine how potential profits from lying affect decision time, we conducted two analyses. First, we regressed value to self (ΔV_{self}) onto reaction times, clustering the errors per participant, to get an across-participant measure of the relationship between the two variables. Second, we ran participant-specific correlations of reaction times and value to self, yielding a correlation coefficient per participant. Then, we examined whether this resulting correlation is related to other behavioral measures by correlating it with the overall dishonesty and with self-regarding motive for dishonesty.

We find no significant relationship between reaction times and ΔV_{self} ($\beta\Delta V_{self}$=0.005, *p*=0.48). Furthermore, each participant's correlation coefficient between reaction times and ΔV_{self} is unrelated to their $\beta\Delta V_{self}$ (r(27)=-0.02, *p*=0.89). That is, the relationship between potential profits and reaction times does not predict levels of self-interest. Interestingly, it is negatively linked to overall dishonesty (r(27)=-0.74, *p*<0.001), such that honest participants take longer to choose when they can gain a large profit from lying, while dishonest participants take longer to choose when the potential profit from lying is small.

Supplementary materials: Tables

Table S1. List of payoffs on each trial. $Self Lie and $Other Lie are payoffs to the Sender and Receiver, respectively, if the Sender chooses to Lie. $Self Truth and $Other Truth are payoffs to the Sender and Receiver, respectively, if the Sender chooses to tell the truth. Catch trials, in which the Sender's payoff is the same for both alternatives, are marked with an asterisk.

	**$Self Lie**	**$Other Lie**	**$Self Truth**	**$Other Truth**
	21	7	18	12
	36	10	34	30
	26	3	23	8
	35	2	32	22
	24	4	19	14
	30	13	29	26
	35	13	32	31
	26	6	24	19
	28	9	23	10
	35	2	32	8
	17	3	14	11
	29	10	27	17
	15	3	10	9
	22	7	18	13
	*17	4	17	6
	19	5	14	8
	15	1	10	3
	15	4	11	8
	32	11	28	18
	21	9	18	12
	31	9	19	16
	27	5	17	14
	36	23	29	27
	33	6	27	9
	28	1	17	5
	23	17	19	18
	28	12	25	24
	24	8	12	10
	42	18	33	26
	19	4	11	7
	19	3	17	8
	24	9	21	10
	35	14	33	26
	16	1	12	5
	16	3	11	4
	33	12	28	27
	*38	15	38	23
	34	13	33	22
	38	8	33	19
	35	13	30	23
**mean**	**26.925**	**8**	**22.45**	**15.2**
**SD**	**7.74**	**5.26**	**8.21**	**8.06**
**minimum**	**15**	**1**	**10**	**3**
**maximum**	**42**	**23**	**38**	**31**

Supplementary Table S2. Self-interest and Regard-for-others coefficients with and without an interaction term. Greyed out are the excluded participants' results. w/ = with. *p<.05 **p<.01 ***p<.001

SELF-INTEREST		REGARD-FOR-OTHERS		INTERACTION
original (linear)	w/ interaction	original (linear)	w/ interaction	
0	0	0	0	0
0.123***	0.088***	-0.019	-0.066**	-0.134***
0.065	0.023	-0.148**	-0.206***	-0.166**
0.088	0.043	-0.180***	-0.242***	-0.176*
0.169***	0.151**	-0.129**	-0.153**	-0.070
0.120*	0.101*	-0.167***	-0.193***	-0.074
0.065	0.019	-0.229***	-0.291***	-0.178*
0.113*	0.104	-0.142**	-0.155**	-0.032
0.070	0.054	-0.127**	-0.149**	-0.061
0.258***	0.224***	-0.182***	-0.228***	-0.133*
0.204***	0.161**	-0.216***	-0.274***	-0.165*
0.232***	0.196***	-0.182***	-0.231***	-0.140*
0.210***	0.200***	-0.162***	-0.175***	-0.039
0.148**	0.148**	-0.118*	-0.117*	0.003
0.149***	0.123**	-0.174***	-0.210***	-0.104
0.207***	0.188**	-0.221***	-0.247***	-0.073
0.171***	0.159**	-0.193***	-0.209***	-0.047
0.241***	0.238***	-0.176***	-0.180***	-0.012
0.222***	0.203***	-0.232***	-0.258***	-0.075
0.192***	0.192***	-0.207***	-0.207***	0.001
0.289***	0.291***	-0.150***	-0.147***	0.010
0.165***	0.152**	-0.172***	-0.190***	-0.050
0.163***	0.158**	-0.144**	-0.151**	-0.021
0.192***	0.185***	-0.222***	-0.232***	-0.026
0.232***	0.232***	-0.236***	-0.236***	0.001
0.195***	0.187***	-0.281***	-0.291***	-0.030
0.228***	0.249***	-0.090*	-0.062	0.080
0.208***	0.231***	-0.066	-0.035	0.089
0.161**	0.192***	-0.170***	-0.128*	0.121
0.116**	0.109**	0.009	0	-0.026
0.124**	0.127**	-0.015	-0.011	0.010
0.120**	0.117**	0.043	0.039	-0.012
0.069	0.050	0.042	0.017	-0.069

Supplementary Table S3. Results of regression model containing efficiency and inequality, alongside value to self and value to other. Greyed out are the excluded participants' results. * p<.05 ** p<.01 *** p<.001

VERSION 1				VERSION EFFICIENCY' & INEQUALITY'			
**Self interest**	**Regard for other**	**Efficiency**	**inequality**	**Self interest**	**Regard for other**	**Efficiency**	**inequality**
0	0	0	0	0	0	0	0
0.018	0.097*	0.188**	0.002	0.125***	-0.012	-0.001	-0.002
-0.154*	0.0660	0.379***	0.038	0.092	-0.096	-0.004	-0.037
-0.101	0.0147	0.332**	0.020	0.103	-0.161*	0.001	-0.028
0.032	0.0230	0.246*	0.003	0.192***	-0.087	-0.002	-0.033
-0.024	-0.052	0.234	0.055	0.133*	-0.161*	0.003	-0.030
-0.153	0.0036	0.386**	0.015	0.050	-0.284***	0.009	0.002
0.011	-0.099	0.143	0.082	0.166**	-0.030	-0.010	-0.071*
-0.030	-0.088	0.139	0.085	0.105*	-0.025	-0.014**	-0.028
0.094	0.0283	0.311**	-0.030	0.235***	-0.247***	0.009	0.019
-0.036	0.0493	0.431***	0.004	0.188**	-0.251**	0.004	0.018
0.098	-0.056	0.228*	0.028	0.213***	-0.228***	0.005	0.021
0.109	-0.013	0.203*	-0.041	0.216***	-0.153**	0.000	-0.011
0.141	-0.092	0.022	-0.021	0.175**	-0.069	-0.002	-0.043
0.053	-0.111	0.149	0.053	0.154**	-0.164*	-0.001	-0.006
0.028	-0.073	0.293*	0.062	0.191**	-0.256**	0.003	0.021
0.069	-0.072	0.188	-0.008	0.149**	-0.254***	0.008	0.019
0.232**	-0.167	0.016	0.001	0.260***	-0.145*	-0.001	-0.033
0.124	-0.093	0.194	-0.033	0.214***	-0.267***	0.006	0.000
0.121	-0.088	0.151	-0.045	0.169**	-0.260***	0.005	0.028
0.273***	-0.140	0.024	0.009	0.294***	-0.147**	0.001	-0.013
0.150	-0.173	0.017	0.021	0.198***	-0.122	-0.001	-0.058*
0.110	-0.087	0.094	0.002	0.184**	-0.108	-0.002	-0.032
0.145*	-0.211*	0.061	0.047	0.195***	-0.216**	0.000	-0.005
0.216**	-0.246**	0.013	0.032	0.229***	-0.234***	-0.002	0.009
0.182*	-0.244**	0.036	-0.026	0.189***	-0.291***	0.000	0.009
0.265**	-0.159	-0.082	0.032	0.237***	-0.075	-0.001	-0.013
0.229*	-0.181	-0.091	0.106*	0.237***	-0.014	-0.003	-0.043
0.315***	-0.302**	-0.255*	-0.047	0.163**	-0.157*	-0.003	0.004
0.116	-0.026	-0.020	0.040	0.134**	0.050	-0.004	-0.021
0.170*	-0.095	-0.099	0.033	0.161***	0.070	-0.008	-0.045
0.120	0.032	-0.006	0.013	0.139**	0.097	-0.007	-0.015
0.068	0.036	-0.003	0.008	0.077	0.069	-0.004	-0.006

Supplementary materials: Figures

Figure S1. Self and Other payoffs. Potential losses to the Receiver (value to other) on the y-axis and potential gains for the Sender (value to self) on the x-axis. Each dark square represents a unique trial in our experiment.

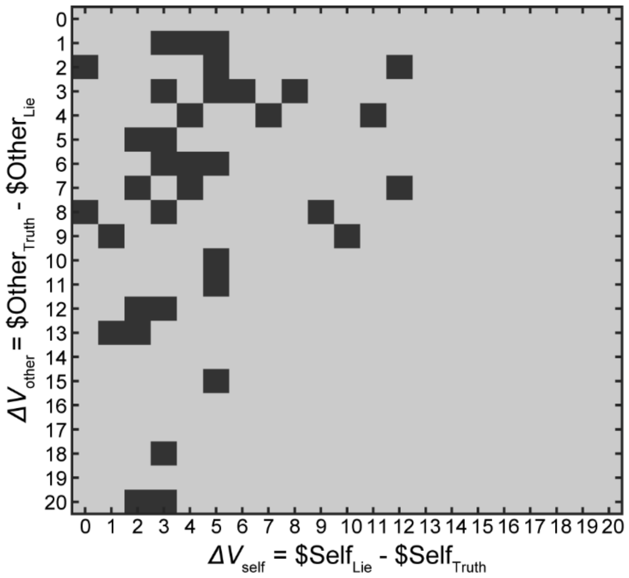

Figure S2. Self-interest and regard for others are not correlated. Coefficient of potential losses to the Receiver (value to other) on the y-axis, and coefficient of potential gains for the Sender (value to self) on the x-axis. Each circle represents a subject. N=28.

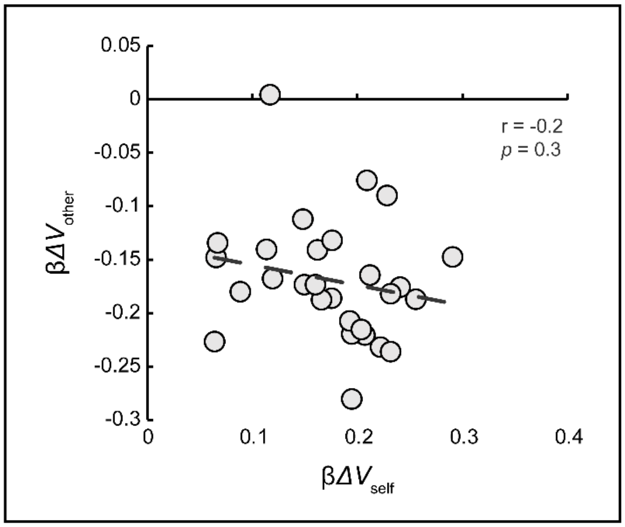

Figure S3. Lie vs. Truth brain map. Contrasting trials in which subjects chose Deceitful message with trials in which they chose the Truthful message. Map thresholded at p=0.001, cluster-size corrected. N=27.

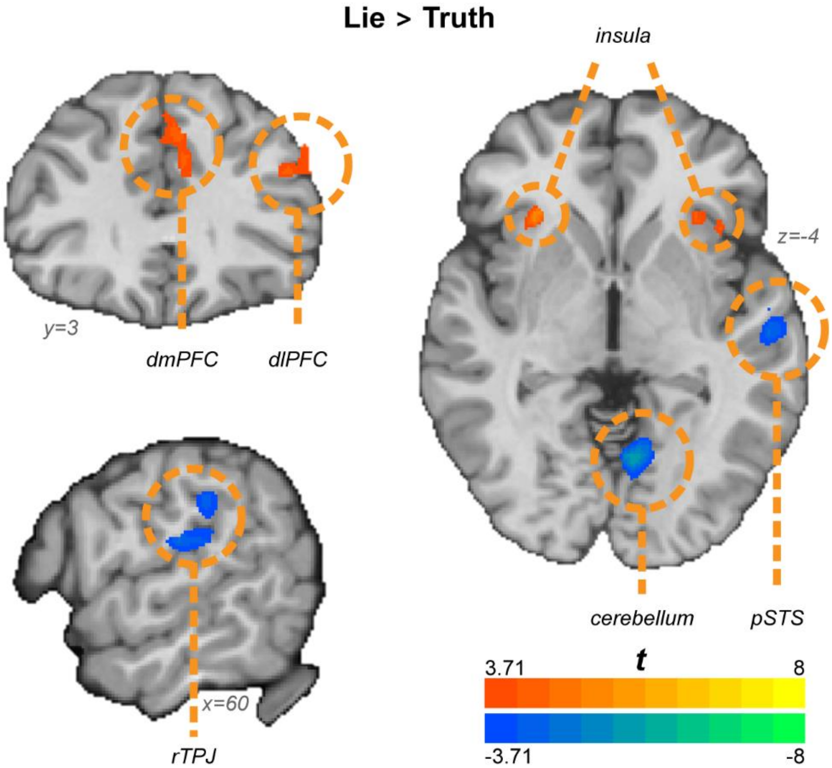

Supplement: Supplementary file 1 — Supplementary file1 [file 41598_2020_72255_MOESM1_ESM.pdf]
